# Topological Darkness: How to Design a Metamaterial for Optical Biosensing with Ultrahigh Sensitivity

**DOI:** 10.1021/acsnano.3c06655

**Published:** 2023-09-22

**Authors:** Gleb I. Tselikov, Artem Danilov, Victoria O. Shipunova, Sergey M. Deyev, Andrei V. Kabashin, Alexander N. Grigorenko

**Affiliations:** † Aix Marseille University, CNRS, UMR 7341 CNRS, LP3, Campus de Luminy−case 917, 13288 Marseille Cedex 9, France; ‡ Shemyakin−Ovchinnikov Institute of Bioorganic Chemistry, Russian Academy of Sciences, 16/10 Miklukho-Maklaya St, Moscow 117997, Russia; § Department of Physics and Astronomy, University of Manchester, Manchester M13 9PL, U.K.; ⊥ MEPhI, Institute of Engineering Physics for Biomedicine (PhysBio), 115409 Moscow, Russia

**Keywords:** topological darkness, scissor effect, topological photonics, dark metamaterials, optical biosensing, ultrasensitive label-free detection

## Abstract

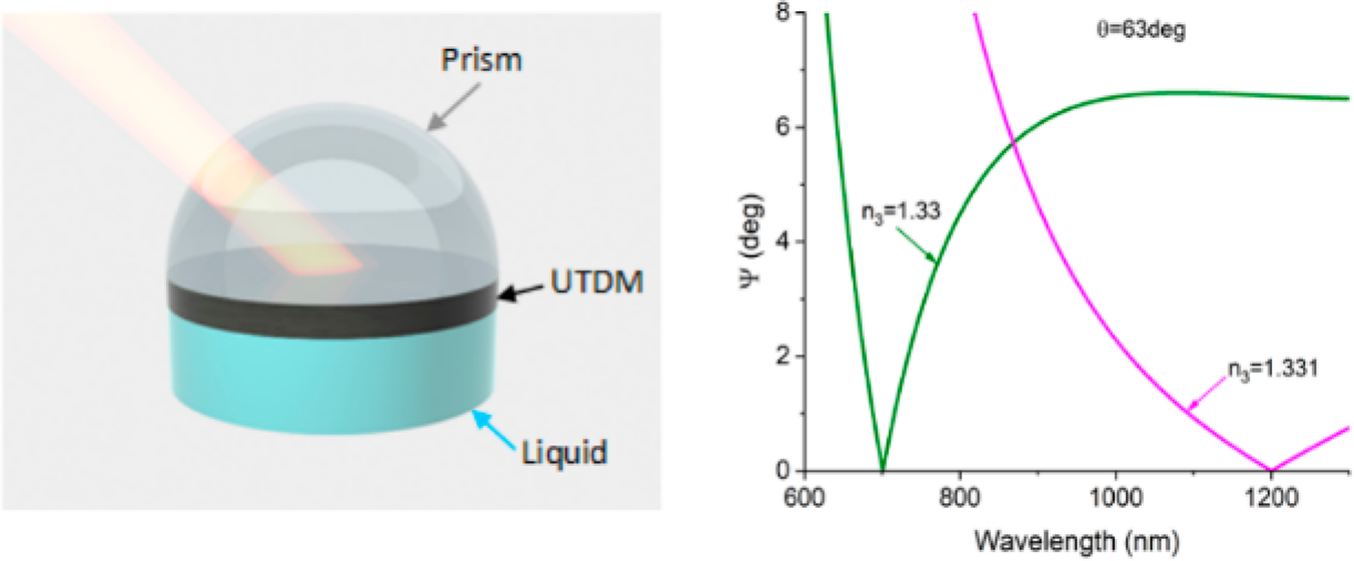

Due to the absence of labels and fast analyses, optical biosensors promise major advances in biomedical diagnostics, security, environmental, and food safety applications. However, the sensitivity of the most advanced plasmonic biosensor implementations has a fundamental limitation caused by losses in the system and/or geometry of biochips. Here, we report a “scissor effect” in topologically dark metamaterials which is capable of providing ultrahigh-amplitude sensitivity to biosensing events, thus solving the bottleneck sensitivity limitation problem. We explain how the “scissor effect” can be realized via the proper design of topologically dark metamaterials and describe strategies for their fabrication. To validate the applicability of this effect in biosensing, we demonstrate the detection of folic acid (vitamin important for human health) in a wide 3-log linear dynamic range with a limit of detection of 0.22 nM, which is orders of magnitude better than those previously reported for all optical counterparts. Our work provides possibilities for designing and realizing plasmonic, semiconductor, and dielectric metamaterials with ultrasensitivity to binding events.

## Introduction

The analysis of affinity binding interactions between a target analyte (e.g., antigen, protein, peptide, DNA, RNA segments) from a biological sample solution and its selective receptor (e.g., antibody, protein, peptide, etc.) immobilized on the surface presents one of key tasks in biomedical diagnostics (e.g., the detection of biomarkers of infections and cancers, cardio control, immune status, rational drug design), environmental and food safety (e.g., the monitoring of toxins or pathogens), and security applications.^1^ Conventional label-based biosensing, currently used in hospitals and laboratories, implies the use of fluorescence or radio labels to mark analytes and thus reports a biomolecular binding, but this approach is insufficiently precise due to the presence of a reaction-interfering labeling step, costly in terms of required laboratory installations, and excessively long. An alternative is offered by optical transduction biosensing to record biomolecular interactions via the monitoring of the optical refractive index (RI) associated with the increase of biolayer thickness, which enables one to immediately report the result of binding and obtain kinetic constants within minutes.^2^ A paramount importance of such a label-free approach was greatly magnified by the recent pandemic, which revealed a critical lack of reliable, easy-to-use, and mass-scale biochips that could give immediate accurate testing results. Profiting from medium-dependent optical excitation of free electron oscillations (plasmons) and a much enhanced plasmon-mediated electric field probing RI variations, plasmonic sensors form the core of label-free biosensing technology applied for the detection of a variety of critically important analytes.^2−4^ However, currently available plasmonic biosensing architectures based on spectral (or angular) interrogation under surface plasmon resonance (SPR) analytes^2,3^ or localized plasmon resonance (LPR)^4^ have a major sensitivity bottleneck. Indeed, the sensitivities of SPR and LPR sensing schemes in terms of the spectral shift per bulk refractive index unit (RIU) change are on the order of (3–10) × 10^3^ ^3^ and (2–5) × 10^2^ nm/RIU,^4^ respectively, which conditions an order of magnitude inferior limit of detection (LOD) compared to label-based sensors. Such a bottleneck is related to a series of fundamental limiting factors, including high losses in plasmonic metals,^5^ low quality of resonances in the case of uncoupled LPRs,^4,6^ and structure geometry limitations in the case of advanced surface lattice resonances (SLR) over periodic nanoparticle arrays.^7,8^ The sensitivity handicap of plasmonic biosensors can be fully compensated by using phase as a sensing parameter instead of spectral interrogation due to the presence of a sharp jump in the very minimum of a resonant curve,^9−11^ but the implementation of ultrasensitive phase interrogation schemes requires properly designed plasmonic architectures and more complicated instrumental readouts.

We recently described a phenomenon referred to as topological darkness (TD),^12,13^ which provides exactly zero light reflection/transmission from a dedicated optical system and is typically observed as a well-defined feature in the measured spectrum consisting of a drop in reflection/transmission with a point of zero light intensity. The absence of reflection/transmission is topologically protected under TD by spectral properties of optical constants of materials and the positioning of the zero reflection/transmission surface and survives any imperfection in the fabrication of topologically dark structures (in the absence of diffuse scattered light). We then described a set of metamaterials (heterostructures^14^ and nanostructures^12,15^) that possess TD and demonstrated that the generation of TD can result in extreme singularities of the phase of light, which could be used in phase interrogation schemes to radically improve the sensitivity of plasmonic label-free biosensors^12,16,17^ as well as to realize plasmonic phase imaging.^9,18^ It is worth noting that exactly zero reflection and phase singularities are important for many applications. They were previously observed for a Brewster geometry,^19^ a Salisbury screen,^20^ a generalized Brewster effect,^21^ optical Tamm states,^22^ strong coupling,^23^ perfect absorbers,^24^ and some other systems without addressing the point of topological protection to imperfections arising during fabrications.

Here, we further explore optical phenomena associated with the generation of TD in designed metamaterials focusing on the spectral response of the TD feature to refractive index variations in the amplitude interrogation channel. We report a “scissor effect” and theoretically show that this effect could yield ultrahigh spectral sensitivity in the recording of binding biosensing events using the standard intensity measurements. We explain how to design and fabricate topologically dark metamaterials (TDMs) for the implementation of the “scissor effect”. To justify the applicability of such metamaterials in biosensing, we performed a label-free quantitative detection of vitamin folic acid (molecular weight 441.4 Da) as an example of a very small molecule that is important for human health. We show that TDMs are capable of monitoring folic acid in a wide range of concentrations (5–5000 nM), while the observed limit of detection (LOD) for TDM of 0.22 nM was orders of magnitude better than those of previously used label-free and label-based methods. Our work provides possibilities for the development of fast, inexpensive, and ultrasensitive label-free optical biosensors which could be based on plasmonic, semiconductor, or dielectric materials and structures.

## Results and Discussion

### Topological Darkness in Attenuated Reflection Geometry

**Figure 1:**
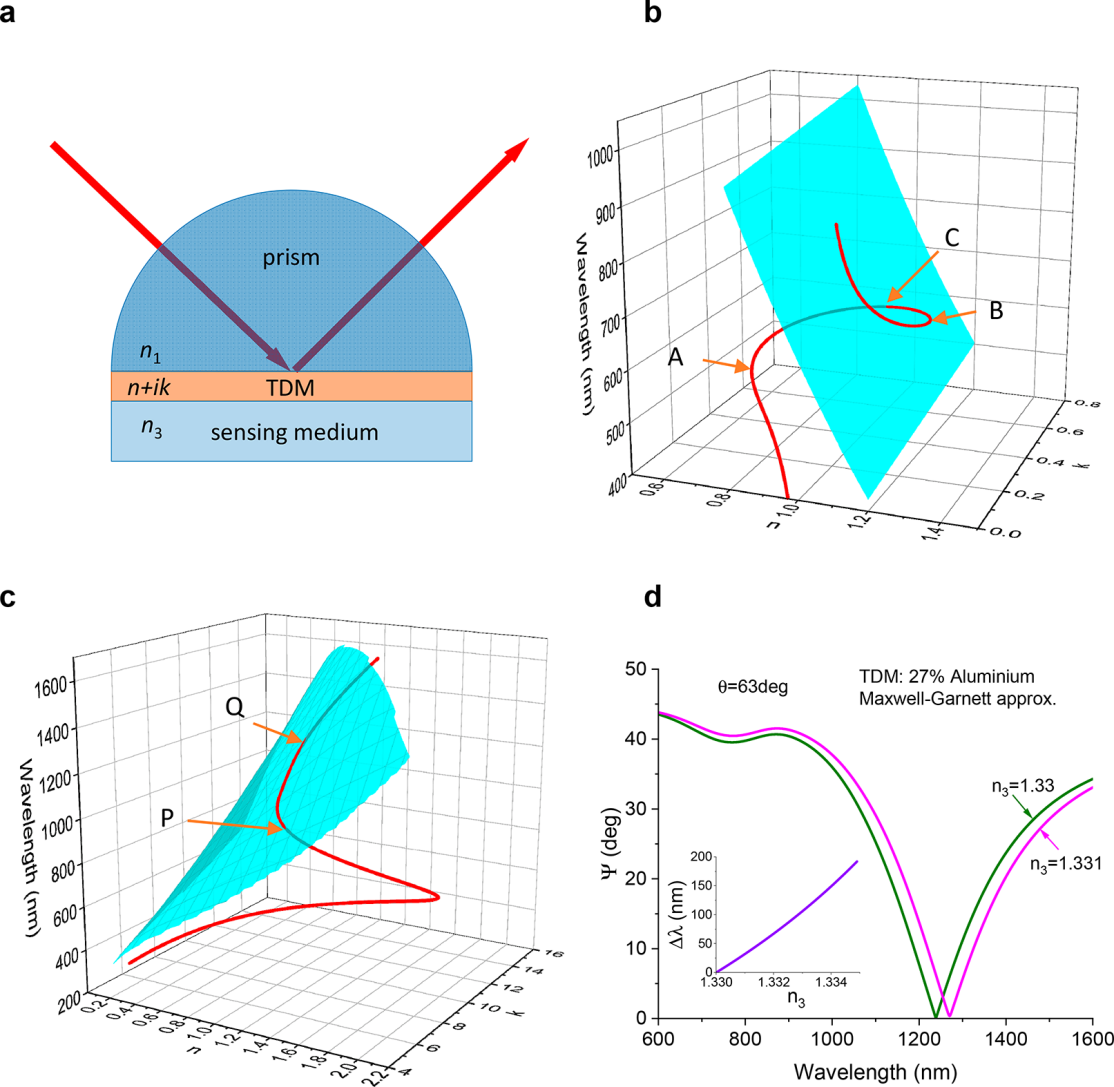
Topological darkness in ATR geometry. (a) The studied structure that consists of a coupling prism, a TDM layer, and a sensing medium (e.g., water of PBS buffer). (b) Topologically protected darkness in the studied structure which is observed at point C where the spectral optical constant curve of TDM (*n*(λ), *k*(λ)), shown in red, intersects the zero reflection surface, shown in cyan. (c) The zero reflection surface (shown in cyan ) calculated for a structure of (a) for TDM of thickness of 21.696 nm, *n*_1_ = 1.513, and *n*_3_ = 1.33. The red curve shows the spectral optical constant curve calculated for a TDM layer made of 27% Al and 73% bottom medium in the Maxwell–Garnett approximation. The red curve intersects the zero reflection surface at a small angle for point Q. (d) Change in the spectral ellipsometric reflection Ψ calculated for two different refractive indices of probed medium *n*_3_ = 1.33 and *n*_3_ = 1.331 (see Methods for the definition of Ψ) at a fixed angle of incidence θ = 63.005° for the point Q of (c). The inset shows the spectral (amplitude) sensitivity of the TD as a function of the refractive index of the bottom medium. The thickness of the TDM layer is 21.696 nm. The thickness of the sample and angle of incidence were rounded to the third digit after the decimal point and resulted in the reflection at the point of darkness at the level of 10^–10^.

We start by recalling the main features of the phenomenon of topological darkness, which guarantees zero light intensity of light reflection (or transmission) at some angle of incidence and some wavelength for a dedicated structure. Here, we concentrate on TD in attenuated reflection geometry (ATR), which is the best suited for biosensing applications.^3^ Let us consider light reflection from a structure shown in Figure 1a, where a thin TDM layer (which can be flat, nanostructured, or heterostructured) is placed at the bottom of a prism with refractive index *n*_1_ and is in contact with a studied medium of refractive index *n*_3_ (which can be water or phosphate-buffered saline buffer often used in biosensing). For a given light polarization, the thickness of the TDM layer, the angle of incidence θ and light wavelength λ, and light reflection from the structure shown in Figure 1a can be made exactly zero by adjusting values of the optical constants (*n*(λ), *k*(λ)) of the TDM layer due to the nature of Fresnel coefficients for the whole structure.^12^ Here,  is the complex refractive index of the TDM layer and ε(λ) is the optical permittivity of the layer.

Hence, for a fixed TDM thickness and an angle of incidence, we will obtain a curve of exactly zero reflection for the studied structure in the 3D space of (*n*(λ), *k*(λ), λ). If we allow the angle of incidence θ to change, we will obtain a zero reflection surface shown in Figure 1b for a hypothetical material (the cyan surface). If the spectral curve of the optical constants for the TDM layer (*n*(λ), *k*(λ)) shown in Figure 1b by the red curve has two points from each sides of the zero reflection surface (see points A and B in Figure 1b), then we will always have an intersection of the spectral curve of TDM optical constants with the zero reflection surface, which would guarantee a zero reflection point C (Figure 1b) due to topology and the Jordan–Brouwer theorem.^12,25−27^ The zero reflection point C is topologically protected in a sense that small imperfections in TDM fabrication will not change the relative positions of points A and B with respect to the zero reflection surface, and hence, the zero reflection point C will be still observed due to the Jordan–Brouwer theorem^12,25,26^ albeit at a slightly different wavelength and angle of incidence. (Topological darkness is an exact phenomenon at which reflection goes to exact zero and the phase of light demonstrates an exact Heaviside π-jump. This implies that the parameters at which TD is observed are described by real numbers. For the sake of simplicity, we will round these numbers to the third digit, which guarantees the intensity reflection at the point of darkness at the level of 10^–10^ at this level of rounding.)

We have to stress that these considerations are only valid when diffuse scattering is small, and one can apply Fresnel theory to the studied structure. The same considerations can also be applied for light transmission and guarantee topologically protected zero transmission points. Here we will concentrate on TD under ATR reflection due to the effectiveness of this geometry in biosensing applications. We will consider the amplitude interrogation and concentrate on the spectral sensitivity of TD. (In parentheses we note that one can use a grating instead of a prism in order to realize ATR geometry. In the presence of gratings, however, we move from the realm of Fresnel materials with defined reflection and transmission to the realm of Fourier metamaterials,^17^ where diffractive beams are present. We will address this case elsewhere.)

### Sensitivity of TD Materials to Biosensing and the Scissor Effect

As we explained above, topological darkness is observed when a zero reflection surface (the cyan surface in Figure 1b) is intersected by an optical constant curve (the red curve in Figure 1b) yielding a zero reflection point C. This point presents a well-defined feature in the reflection spectrum (total darkness!) and could be used for a label-free detection of biological binding events. In biosensing experiments, the refractive index of the sensing medium *n*_3_ will change. This will change the position of the zero reflection surface while the optical constants of the TDM remain the same. Therefore, an intersection of the zero reflection surface and the optical constant curve will happen at a different point and TD will occur at a different wavelength. (Analogous considerations can be applied to the case where a TDM layer is functionalized to provide selective detection of bio-objects. In such a scenario, bioreactions will modify the properties of this functionalized layer that would result in a shift of the zero reflection surface.) The sensitivity of TDM amplitude detection then can be expressed in terms of *S* = Δλ/Δ*n*_3_, where Δλ is the spectral shift of the zero reflection point caused by the change in the refractive index of the sensing medium Δ*n*_3_. It appears that this sensitivity should not be large, as the shift of the zero reflection surface is normally small. However, in contrast to biosensing based on LPR or SPR platforms, where one measures a shift of a resonance curve with respect to its original position (which happens due to biological binding events in the probed medium or at the surface of biochip), *in the case of TD we measure how the zero reflection surface is moving with respect to the spectral curve (n(λ), k(λ)) of TDMs*.

As a result, the sensitivity of TD structures crucially depends on the angle, α, at which the spectral curve of TDM optical constants intersects the zero reflection surface. In the Supporting Information we show that the sensitivity can be written as , where *Q* is some constant which depends on the geometry and optical constants of the structure. Assume now that the thickness of TDM is fixed, which fixes the position of the zero reflection surface. Then, by changing the optical constant spectral curve of TDM we can achieve different angles of intersection, α*.* The smaller the angle of intersection is achieved, the greater the spectral sensitivity will be. At small angle α, the sensitivity can be written as *S* = *Q*/α, where α is expressed in radians and could be very large. We will refer to the large increase of sensitivity due to small intersection angle as the “scissor effect” following a scissor analogy, where the point of intersection of scissor blades moves much faster than the blades themselves with an increase of the speed being proportional to 1/ϕ, where ϕ is the angle between blades (at small angles ϕ).

It is easy to find a metamaterial for which a spectral curve of optical constants (*n*(λ), *k*(λ)) intersects the zero reflection surface at a small angle. Figure 1c shows a theoretical example of such a situation for a structure depicted in Figure 1a. In this case, a TDM of thickness *d* = 21.696 nm is produced by a mixture of Aluminum (27%) and the bottom medium (73%) described by Maxwell–Garnett theory^28^ (a particular type of effective medium is not important, as this effect will be observed for, e.g., Bruggeman’s effective medium^28^ and others). This TDM has spectral optical parameters (*n*(λ), *k*(λ)) shown in Figure 1c by the red curve. The zero reflection surface for this TDM structure is shown in Figure 1c by the cyan surface. We see that the spectral curve indeed intersects the zero reflection surface at a small angle for the point Q of zero reflection (it happens at the angle of incidence θ = 63.005° and wavelength 1238.8 nm). By calculating the shift of the zero reflection surface induced by a small change of the index of refraction of the studied medium, we can find a point Q′ of intersection of the spectral curve with the shifted zero reflection surface which gives us a shifted spectral position of zero reflection and a slightly different angle of incidence at which TD happens. This calculation yields high spectral sensitivity of *S* ≈ 6 × 10^3^ nm/RIU (where RIU is the refractive index unit) for the point Q of the discussed structure for the case where the angle of incidence is allowed to change in order to restore the TD.

It should be noted, however, that the angle of light incidence is often fixed in optical interrogation schemes. In this case, instead of a zero reflection surface (which is responsible for TD at varied angles of incidence), we are dealing with zero reflection lines corresponding to a particular angle of incidence. The scissor effect is still present in this case. However, it will be conditioned by an angle between a zero reflection line corresponding to the fixed angle of incidence and the optical constant curve. Unexpectedly, the sensitivity of the TD metamaterials can be even larger for the case of fixed-angle interrogation. (It happens because in this case the sensitivity will be defined by the shift of the reflection minimum instead of the shift of the TD position.) For example, the Maxwell–Garnett TDM layer consisting of 27% of aluminum and 73% of the bottom medium shows even higher spectral sensitivity of *S* ≈ 3.2 × 10^4^ nm/RIU which can be deduced from Figure 1d and its inset that depicts the shift of the spectral position of the reflection minimum caused by the changes of the refractive index *n*_3_ of the sensing medium (a definition of ellipsomertic reflection used in Figure 1d is provided in Methods) at a fixed angle of incidence θ = 63.005°. How this higher spectral sensitivity (which is comparable with SPR sensitivity of gold chips at this spectral range^3^) is connected to “spoof” SPRs will be discussed in future publications.

### Design of Ultrasensitive Topologically Dark Metamaterials

**Figure 2:**
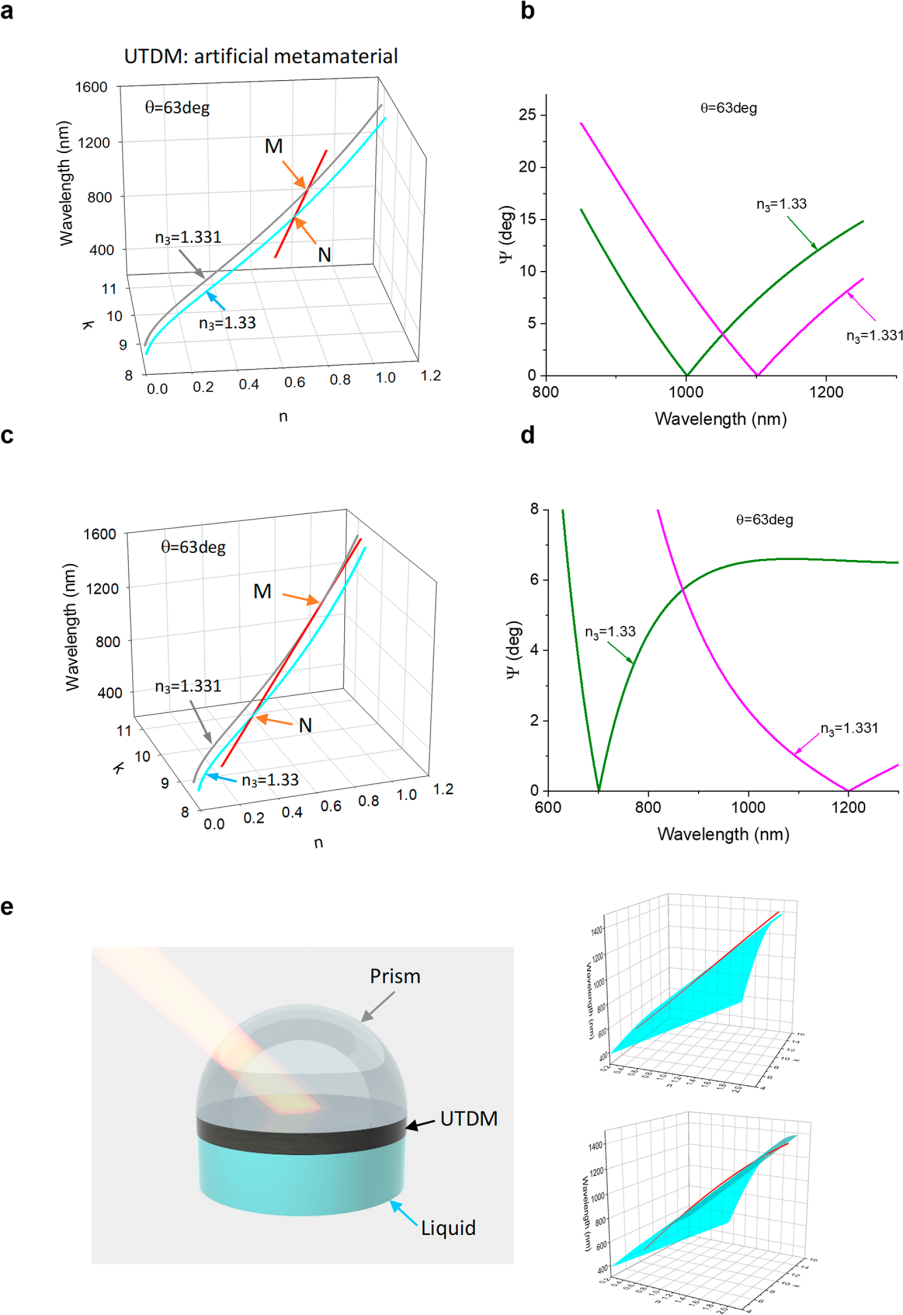
Unlimited spectral sensitivity of topological dark metamaterials in biosensing. (a) Zero reflection curves for two different refractive indices *n*_3_ of sensing medium shown in cyan and gray observed for the angle of incidence 63° and the TDM thickness of 21.696 nm. The red curve shows a spectral constant curve of a hypothetical metamaterial that connects points M and N. (b) The ellipsometric reflection Ψ calculated for two different refractive indices of probed medium *n*_3_ = 1.33 and *n*_3_ = 1.331 for TDM described by the red curve of (a) which shows a shift of the zero reflection point by 100 nm. (c) Same as for (a) with a larger spectral distance between points M and N. (d) Same as for (b) with a larger shift of the zero reflection point by 500 nm. (e) The optics and geometry of UTDM. The top and bottom insets on the right show typical scenarios at which UTDM is observed, where the cyan surface represents the zero reflection surface and the red curve represents the spectral curve of the optical constants of UTDM.

The scenario described in Figure 1c gives a simple theoretical algorithm for *designing ultrasensitive TDM (UTDM) with ultrahigh spectral sensitivity to sensing medium* for a structure of Figure 1a operating at a fixed angle. This algorithm is illustrated in Figure 2. Step 1: fix the thickness of the TDM layer and the angle of incidence. (One can select TDM thickness at 5–30 nm to reduce the number of TD points, and the angle of incidence θ > arcsin(*n*_3_/*n*_1_) in order to realize ATR, in which only evanescent waves are present in the sensing medium.) For example, for Figure 2 we selected the TDM thickness *d* = 21.696 nm and the angle of incidence θ = 63°. Step 2: construct zero reflection curves at these parameters for two different refractive indices *n*_3_ (these curves are shown in Figure 2a for n′_3_ = 1.33 and *n*″_3_ = 1.331 by the cyan and gray, respectively). Step 3: choose two points on these two zero reflection curves separated by a large enough spectral distance Δλ, e.g., points M and N of Figure 2a. Step 4: design a metamaterial for which the optical constant spectral curve passes through the points M and N as shown in Figure 2a by the red curve. By design, this metamaterial will show exactly zero reflection for both refractive indices of the sensing medium separated by the chosen spectral distance (see Figure 2b). Hence, the constructed TDM will have spectral sensitivity *S* = Δλ/Δ*n*_3_ which can be, in principle, as large as required, in accordance with the “scissor effect”. Figure 2b plots two reflection curves for the structure of Figure 1a with UTDM spectrally, as described by the red curve of Figure 2a. We observe a large shift of the zero reflection wavelength at a small change of the refractive index of sensing medium, which results in a TD spectral sensitivity of *S* = 10^5^ nm/RIU.

To demonstrate that UTDM sensitivity can be of any given large number, Figure 2c shows an application of the same recipe with a much larger spectral separation of points M and N. Again, we need to design a metamaterial with the spectral curve of optical constants that pass through the points M and N shown as the red curve of Figure 2c. This metamaterial yields an even larger wavelength shift of 500 nm for the zero reflection point at the change of the refractive index of sensing medium Δ*n*_3_ = 0.001 resulting in a sensitivity of *S* = 5 × 10^5^ nm/RIU (see Figure 2d). This sensitivity is about 2 orders of magnitude higher than that observed for optimized gold chips under SPR^3^ at the same wavelengths. It clear that the suggested algorithm could provide a “theoretical” metamaterial with basically any given number of spectral sensitivity. Such high amplitude sensitivity could be used for realizing ultrasensitive label-free biosensing with a simple readout even in mobile phones.

### Strategies to Achieve the Scissor Effect

Figure 2e summarizes the main features of UTDM biosensing in the case of the simple structure shown in Figure 1a, which comprises a coupling prism, a thin functionalized TDM layer, and sensing medium. The light falls on the structure at the angle of incidence, which is larger than the critical angle of the structure, θ_c_ = arcsin(*n*_3_/*n*_1_), producing only evanescent fields in the sensing medium (light transmission through the sensing medium is absent). The reflection from the structure is also absent due to TD. Hence, TDM behaves as a perfect absorber in this case.^29,30^ To achieve the “scissor effect” and high biosensitivity, the spectral curve of UTDM should either intersect the zero reflection surface at a very small angle (as shown in the top inset of Figure 2e) or almost touch it by crossing in two close points (see the bottom inset of Figure 2e), leading to large spectral changes of the TD point at small changes of the sensing medium and providing the wherewithal to design different types of UTDM. Finally, the small angle of intersection between the spectral curve and the zero reflection surface implies a small window of angles at which UTDM works, which explains why this effect was not discussed before.

An important stage of designing UTDM is based on the possibility to fabricate a metamaterial with given spectral parameters (*n*(λ), *k*(λ)). This was a subject of intense studies in recent years driven by advances in nano-optics and plasmonics.^31−36^ There are several approaches to design tailor-made metamaterials with a given spectral curve based on natural resonances,^12^ complex nanostructuring,^12^ SPR heterostructures,^14^ etc. These metamaterials can be based on plasmonic, semiconductor, or dielectric nanoheterostructures.

**Figure 3:**
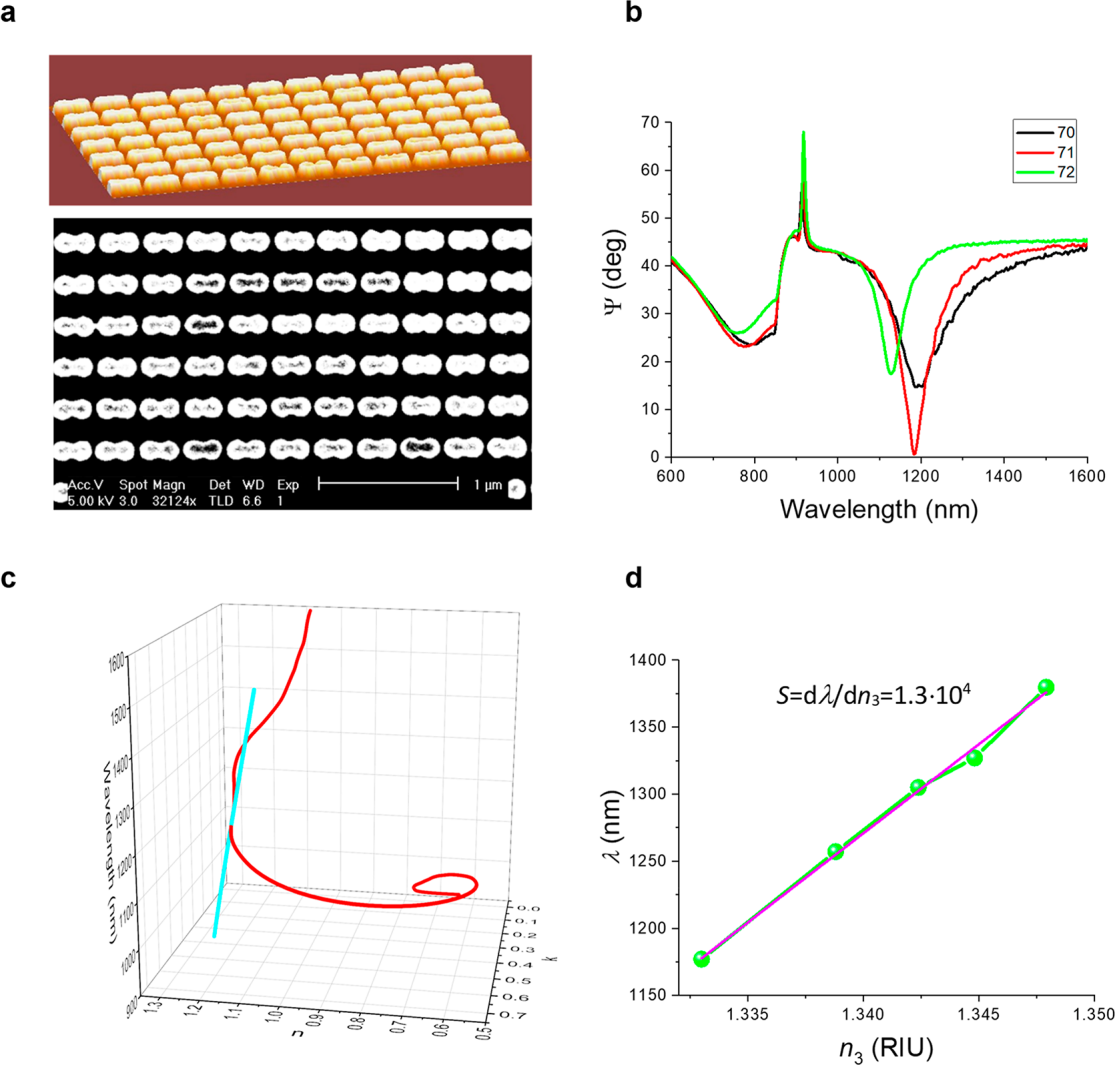
The “scissor effect” and related enhanced spectral sensitivity of a fabricated topologically dark metamaterial based on a regular square array of Au nanoparticles. (a) SEM images showing the fabricated metamaterial. The top is a 3D image, and the bottom is a 2D image. (b) The ellipsometric reflection Ψ measured for three angles of incidence for TDM shown in (a). (c) An intersection of a zero reflection curve calculated at measured parameters (the cyan curve) with the spectral curve of optical constants of TDM extracted from (b). (d) The change of the reflection minimum for the TDM structure of a measured using water–glycerol mixtures see (Methods).

It is quite fortunate and counterintuitive that TDM (showing the “scissor effect”) can be realized with reasonably simple nanostructured metal-dielectric metamaterials, e.g., formed by a regular square array of metal nanoparticles. Figure 3a shows SEM images of a simple TDM produced by a square array of gold dumbbells fabricated on a glass substrate (see Methods) which was used as a TDM layer in the structure of Figure 1a. The measured ATR ellipsometric reflection of the structure with this TDM layer is shown in Figure 3b (see the Supporting Information for the experimental details). This reflection has two main features—an extremely narrow diffraction-coupled surface lattice resonance (SLR) at a wavelength of 930 nm (where *s*-polarized reflection goes close to zero)—and the point of topological darkness was observed at the wavelength of 1185 nm and angle of incidence of 71°. The point of TD was observed in a narrow range of angle of incidences (the change of angle of incidence by 1° was enough to restore a large reflection), which suggests that it can lead to the scenario described in Figure 2e. Figure 3c shows that the curve of spectral constants of TDM extracted from Figure 1b indeed intersects the zero reflection line at the acute angle.

To check the spectral sensitivity of TDM shown in Figure 3a, we measured the experimental change of the reflection minimum by changing the refractive index of sensing medium *n*_3_. This was done by using water–glycerol mixtures (Supporting Information) and resulted in a experimental sensitivity of *S* = 1.3 × 10^4^ nm/RIU, which is more than 2 times higher than SPR sensitivity at these wavelengths^3^ (Figure 3d). It is also worth comparing the sensitivity of the TD mode of the studied TD metamaterial with the sensitivity of SLR modes.^16^ According to the theory, diffraction-coupled SLRs should provide sensitivity to RI variations at the level of Δλ/Δ*n* ≈ *d*, where *d* is the array period.^16^ This yields 320 nm/RIU for the SLR resonance (observed at 930 nm in the studied dumbbell array), which is 40 times smaller than the sensitivity of the TD mode, confirming its different nature. The measured sensitivity of the TD mode hence can compete with phase sensitivity obtained with the help of surface lattice resonances,^8^ hyperbolic metamaterials,^37^ or the hyperbolic Goos–Hanchen effect.^38^

### Ultrasensitive Detection of Folic Acid with the Help of Topologically Dark Metamaterials

**Figure 4:**
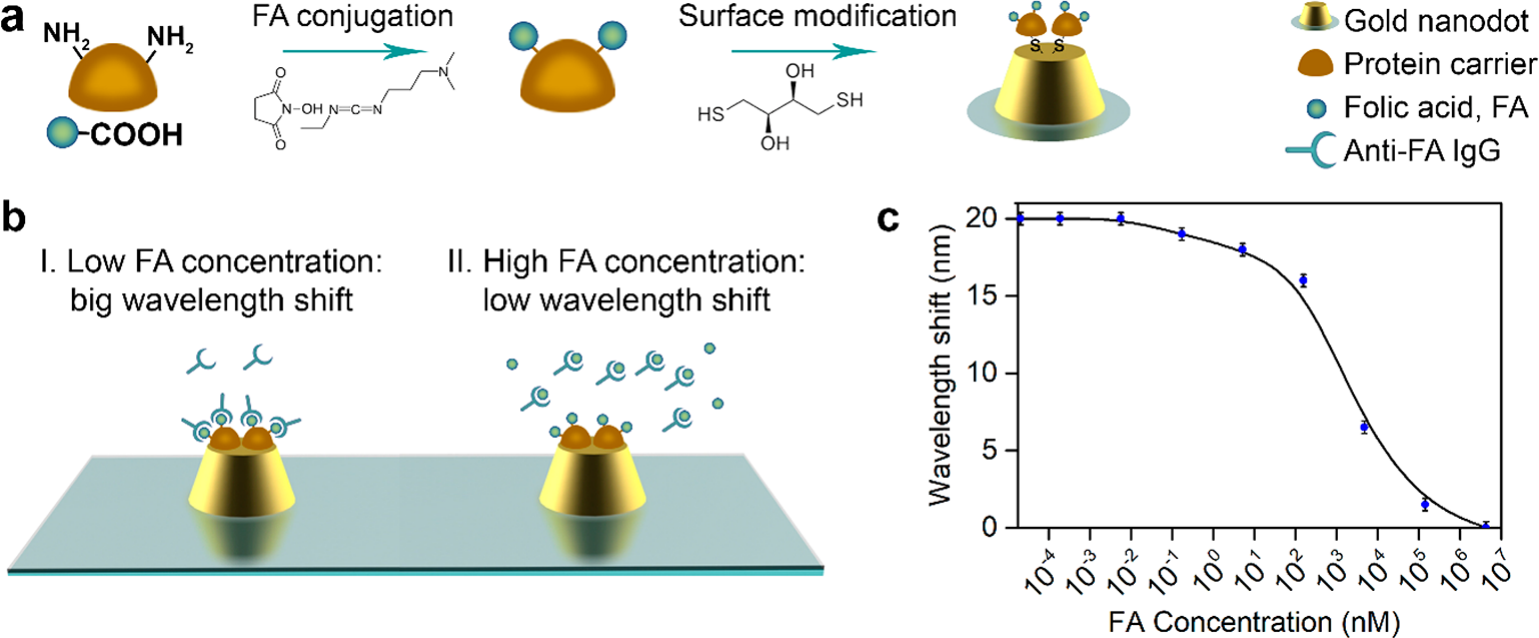
Label-free detection of folic acid using TDM in conditions of the “scissor effect”. (a) Schematic illustration of gold surface modification with carrier protein conjugated to FA for the implementation of competitive FA detection. First, the carrier protein (bovine serum albumin) is conjugated to FA via carbodiimide chemistry. Then, protein-FA is incubated with DTT to reduce SH groups to enable gold surface biomodification. (b) Competitive label-free assay illustration for the detection of FA. A sample under investigation with FA is preincubated with anti-FA IgG. Next, the obtained complexes FA*anti-FA IgG are pumped through the liquid cell with gold nanodots coated with FA. When FA concentration is low (I), all FA binding sites on gold surface are coated with anti-FA antibodies, thus resulting in a sufficiently large detected signal. When the FA concentration is high enough (II), all anti-FAs are preblocked with FA, so binding of IgG to the gold surface is impossible, thus resulting in a small detecting signal. (c) Dependence of the spectral position of the minimum of reflection on FA concentration in nM.

To illustrate the applicability of TDMs in biosensing and the experimental usage of the “scissor effect” in biosensing, we carried out label-free biosensing tests using a protocol for quantitative detection of water-soluble vitamin folic acid (FA, B9, and M) as a prominent example of a low-molecular-weight compound (441.4 Da). Being a parent of a group of enzyme cofactors (referred to as folates), which play a significant role in the formation of purines, pyrimidines, and methionine, FA is involved in DNA, RNA, and protein biosynthesis, while the deviation of its level from normal values (3–20 ng/mL or 6.8–45.3 nM in human serum) can cause major health problems, including anemia, psychiatric disorders, cardiovascular and cerebrovascular diseases, carcinogenesis, or neuronal tube defects in newborns. As the sample serum is typically limited (especially in newborns), the methods for FA diagnosis should be extremely sensitive, while the monitoring of the FA level should be carried out in a relatively wide dynamic range. For these tests, we used a TDM shown in Figure 3, which provides a fairly high sensitivity to bulk RI variations (1.3 × 10^4^ nm/RIU). The detection was implemented in a competitive assay mode, which is beneficial for small analyte such as FA. A schematic of gold surface modification and a related experimental procedure are shown in Figure 4a,b (a more detailed experimental description is presented in the Supporting Information).

In the experiments, FA concentration in the solution pumped through liquid cell was varied between 2 × 10^–5^ and 4 × 10^6^ nM. As shown in Figure 4c, the change of FA concentration led to a gradual spectral shift of the minimum of reflection associated with TD by about 20 nm and at a concentration of 5 pM of FA comes to a saturation. It is important that the sensing response was linear in a wide range of FA concentrations from 5 to 5000 nm (3-log range), which is crucial for tasks of FA monitoring. The limit of detection (LOD) was determined by the 3σ criterion^39^ as follows: LOD = *A* – 3σ, where A is the maximal signal on the saturation and σ is the error in the measurement at the last “zero” point. This method involves measuring the signal (response) of the biosensor in the presence of a low concentration of the analyte and calculating the standard deviation (σ) of the background noise. The LOD is then defined as the concentration of the analyte that produces a signal that is 3 standard deviations above the mean of the background noise. The minus sign in the formula above comes from the competitive nature of the reaction. Substituting the value of σ for spectral measurements (0.4 nm), we found that the spectral LOD was equal to 0.22 nM. Such a level of LOD seems to drastically outperform reported LOD values for all optical biosensor counterparts. Indeed, this value is at least 10 times lower than those reported for label-based (0.38 μM,^40^ 80 nM,^41^ 2.9 nM under nanoparticle-enhanced SPR^42^) and label-free (2.3 nM under SPR^43^ and 100 nM under LPR^44^) sensors, as well as orders of magnitude lower than those reported in most alternative approaches (electrochemical, capillary electrophoresis, etc.).^45^ The LODs for the developed biosensor and previously published studies are summarized in Table 1.

**Table 1:** Parameters of Optical Biosensors for Folic Acid Detection

no.	label	detection principle	limit of detection, nm	reference
1	label-based	fluorescence, carbon quantum dots	380	(29)
2	label-based	fluorescence, CuInS_2_ quantum dots	80	(30)
3	mixed	nanoparticle-enhanced SPR	2.9	(31)
4	label-free	SPR, Biacore	2.3	(32)
5	label-free	LPR, gold nanorods	100	(33)
6	label-free	topological-darkness-based biosensor	0.22	current study

Although we used folic acid as the proof-of-concept analyte for TDM, detecting similar analytes in future studies could enhance the specificity of the biosensor. It is worth noting that the main objective of our study was to demonstrate the feasibility of TDM design and detection methodology, which can be applied to various analytes, including not only small molecules but also DNA, RNA, and proteins. This research can thus contribute to advancing biosensor technology, and further investigations could focus on detecting any type of analyte to improve the specificity of the biosensor without compromising its extreme sensitivity. It is interesting to note that the obtained 10-fold gain of sensitivity compared to SPR^43^ could not be explained solely by the increase of bulk sensitivity to RI variations, as the studied sample exhibited only slightly higher sensitivity (1.3 × 10^4^ nm/RIU as compared to (2–5) × 10^3^ nm/RIU observed under SPR in an analogous spectral range). We suppose that such a gain is due to a stronger electric field probing target molecules, as well as to better localization of electromagnetic fields observed for the case of nanostructured TDM, as compared to a flat gold surface under SPR. It should be noted that the recorded LOD was limited by the spectral sensitivity of TDM (1.3 × 10^4^ nm/RIU) conditioned by the efficiency of “scissor effect” for a concrete experimental layout. As we theoretically showed above, one can design TDMs providing sensitivities 10^5^–10^6^ nm/RIU and higher, which are unimaginable within current optical transduction biosensing technology. It is worth noting that the high sensitivity associated with topological darkness effects described here could already be observed in some previous studies, but these effects were not clearly identified and properly explained. In particular, an anomalously high sensitivity of 3.2 × 10^4^ nm/RIU was reported using plasmonic nanorod metamaterial composed of a “forest” of long Au nanorods (400–450 nm) arranged perpendicularly on a glass substrate,^46^ while a sensitivity of 2.4 × 10^3^ nm/RIU was observed in a 3D woodpile-based plasmonic crystal metamaterial.^47^ A detailed analysis of the experimental data reported in these works shows that in both cases the quasi-effective media provided almost zero intensity in reflection under a relatively narrow range of angle variations, which is the telltale feature of the ultrasensitive TD phenomenon.

### Promising Architecture in Label-Free Biosensing

The main message of the presented study is that an implementation of the “scissor effect” via a proper design of topologically dark metamaterials allows one to propose promising architectures in label-free optical biosensing with ultrahigh sensitivity. Indeed, a conventional paradigm in such biosensing modality implies the usage of resonant phenomena (SPR, LPR, etc.) and following the resonant curve with respect to its initial position due to biological binding events on the sensor surface. Instead, we propose to use the phenomenon of topological zero reflection, realized with the help of a TDM mimicking effective media with appropriate optical constants *n* and *k*, and study the spectral shifts of this zero reflection position with respect to the spectral curve *n*(λ) and *k*(λ) of the TDM. We showed here that under a proper design of TDM, the “scissor effect” conditioned by a very small angle of intersection of the spectral curve and zero reflection surface can be realized, which leads to an orders of magnitude increase of biosensor sensitivity. We also described a pathway on how to design UTDM which relies on the possibility of designing a metamaterial with given spectral properties. It is important that UTDMs can be realized using not only metal but also semiconductor or dielectric structures, while local structure imperfections appear to be not significant due to topologically protected zero reflection. Therefore, in contrast to many other metamaterial structures, they can be fabricated by cost-efficient methods, such as nanoparticle lithography or self-assembling. Our work provides endless opportunities for theoreticians to design hypersensitive TDMs for biosensing (and other applications) as well as exciting possibilities for experimentalists to realize these UTDMs using nanostructured and heterostructured (meta)materials.

In view of potential applications, we demonstrated the possibility of FA monitoring using a TDM, which provided a record sensitivity combined with a linear response in a wide dynamic range. TDMs can be easily adapted for more intelligent fundamental and biomedical applications or for highly sensitive detection of other low-molecular-weight compounds and proteins, including clinically relevant antibiotics, toxins, hormones, and disease-related antibodies. It is also important that UTDMs can be properly structured to offer a sensor surface for the immobilization of multiple receptors, which allows for high-throughput analyses of numerous lead analytes in parallel. For example, ultrasensitive detection of low-molecular-weight compounds (such as various carbohydrate metabolites, homocysteine, cholesterol, vitamins, and various hormones) is necessary in clinical practice for analyzing the current stage and monitoring the dynamics of the disease in patients. The development of reliable and sensitive methods for analyzing illegal drugs or doping in sports is also of high importance. As for the food industry, different methods of analysis with significant sample dilution (designed to eliminate the matrix effect) and without sophisticated sample preparation for the detection of pathogens, antibiotics, or vitamins are also extremely popular. Taking into account the relatively low cost of both UTDMs and required hardware, the UTDM-based biosensing technologies represent an appealing platform for the next-generation express point-of-care testing for, e.g., COVID-19 rapid analysis or intraoperative diagnostics.

## Conclusions

To conclude, we report the “scissor effect” using topologically dark metamaterials, which can provide extremely high (theoretically unlimited) spectral sensitivity in the detection of biological binding events and thus allow one to solve the bottleneck sensitivity limitation problem of current optical label-free biosensing technology. We provide an algorithm for designing ultrasensitive TDMs with any given spectral sensitivity. Using the “scissor effect”, we experimentally demonstrated the detection of folic acid in a wide 3-log linear dynamic range with a limit of detection of 0.22 nM, which is orders of magnitude better than those previously reported for all optical counterparts. Our work could lead to robust, inexpensive, fast, and accessible label-free optical biosensors. It is not clear whether label-free UTDM biosensing can replace label-based methods; however, it has the potential to challenge them.

## Methods

### Device Fabrication

High-quality regular and homogeneous arrays of gold coupled dot pairs were produced by e-beam lithography on a clean microscopic glass substrate covered by a thin Cr (5 nm) sublayer (routinely used to avoid charging during electron beam lithography). We employed a double-layered resist (80 nm of 3% 495 poly(methyl methacrylate) (PMMA) for the bottom resist layer and 50 nm of 2% 950 PMMA for the top layer) in order to improve the subsequent lift-off process. The exposure was performed using a LEO-RAITH e-beam lithography system followed by development in 1:3 methyl isobutyl ketone (MIBK):isopropanol (IPA) developer for 30 s. After lithography, we deposited 5 nm of Cr (to improve adhesion) and 90 nm Au by electron beam evaporation with the help of a Moorfield system. Our deposition rate was controlled precisely at 1.0 Å s^–1^, and the base pressure was 1.0 × 10^–6^ Torr. The thickness of the growing metal film was monitored by a calibrated quartz microbalance (CQM). For the lift-off procedure, the sample was immersed in acetone for approximately 1 h. Finally, a scanning electron microscopy (SEM) image of the fabricated double-nanodot structure was taken to determine the size of dots, periodicity of the nanostructure, and separation between dots in the pair. The fabrication of our samples is described in more detail in our previous works.^12^

### Ellipsometric Parameters Ψ and Δ

Ellipsometry is a sensitive method that can be used to measure the optical properties of materials. Ellipsometry routinely provides the amplitude (Ψ) and phase (Δ) parameters for light reflected from an object. These parameters are related to the complex reflected field amplitudes  and  (where *E*_i_ is the incident light and *E*_p_ and *E*_s_ are the reflected fields for p and s polarizations, respectively) by the equation .^48^ The function Ψ represents the modulus of the ratio of Fresnel reflection amplitudes for p and s polarizations, while Δ provides the phase shift between the p and s components of the light. Hence, Ψ represents an amplitude (intensity) channel of interrogation and Δ represents a phase channel. A spectroscopic ellipsometer can measure the dependence of Ψ and Δ on light wavelength. In addition, a variable-angle ellipsometer allows one to measure the spectral dependences of Ψ and Δ on the angle of incidence. Intensity reflections and transmissions (*R*_p_*, T*_p_) for p and (*R*_s_*, T*_*s*_*)* for s polarized light at various angles of incidence can also be measured. The measurements of TDM nanostructures were performed across a wavelength range of 240–1700 nm with the help of a variable-angle spectroscopic ellipsometer (VASE) M-2000F, manufactured by Woollam, using a rotating compensator-analyzer configuration.

## Data Availability

The data that support the findings of this study are available from the corresponding author upon reasonable request.

## Supporting Information Available

Dependence of TDM sensitivity on intersection angle, sample fabrication procedures, description of ellipsometric parameters, ATR spectroscopic ellipsometry and spectral sensitivity of samples measured with water–glycerol mixtures, and biosensing experiment on quantification of folic acid (PDF)
