# Supplementary material for: Topological Darkness: How to Design a Metamaterial for Optical Biosensing with Ultrahigh Sensitivity
Source: ACS Nano. 2023 Sep 22;17(19):19338–48. doi: 10.1021/acsnano.3c06655 (PMC10569102; doi:10.1021/acsnano.3c06655)
Supplement: Supplementary file 1 — nn3c06655_si_001.pdf [file nn3c06655_si_001.pdf]

Supporting Information for

Topological Darkness: How to Design a Metamaterial for Optical Biosensing with Ultrahigh Sensitivity

Gleb I. Tselikov[1], Artem Danilov[1], Victoria O. Shipunova[2], Sergey Deyev[2], Andrei Kabashin[1], Alexander N. Grigorenko[3*]

[1] *Aix Marseille University, CNRS, UMR 7341 CNRS, LP3, Campus de Luminy – case 917, 13288, Marseille Cedex 9, France*

[2] *Shemyakin–Ovchinnikov Institute of Bioorganic Chemistry, Russian Academy of Sciences, 16/10 Miklukho-Maklaya St, Moscow 117997, Russia*

[3] *Department of Physics and Astronomy, University of Manchester, Manchester, M13 9PL, UK*

* corresponding author: sasha@manchester.ac.uk

Dependence of TDM sensitivity on intersection angle α

Consider a zero reflection surface calculated for a structure of main Fig. 1a which is shown in main Fig 1b (the cyan surface). Let us introduce dimensionless wavelength $\bar{\lambda} = \frac{\lambda}{\lambda_0}$, where λ_0 is some wavelength scale, and replot the zero reflection surface and the optical constant curve (the red curve of Fig. 1b) in the dimensionless coordinates (n,k,$\bar{\lambda}$). At the point of darkness C (main Fig. 1b) we can introduce two unit vectors:

i) the unit vector $\hat{\mathbf{t}} = \left(\frac{dn}{d\bar{\lambda}}, \frac{dk}{d\bar{\lambda}}, 1 \right) \frac{1}{\sqrt{\left(\frac{dn}{d\bar{\lambda}} \right)^2 + \left(\frac{dk}{d\bar{\lambda}} \right)^2 + 1}}$ which is tangential to the optical constant curve in the space of ($n,k,\bar{\lambda}$).

ii) the unit vector $\hat{\mathbf{n}}$ which represents a normal to the zero reflection surface.

It is clear that the angle between the vectors $\hat{\mathbf{t}}$ and $\hat{\mathbf{n}}$ is $\frac{\pi}{2} - \alpha$, where α is the angle of intersection of the spectral curve of optical constants (the red curve of Fig. 1b) with the zero reflection surface (the cyan surface of Fig. 1b). During biosensing experiments, the refractive index of the sensing medium n_3 will change and the zero reflection surface will move by the distance $K\Delta n_3$ in the direction of the normal $\hat{\mathbf{n}}$. (The coefficient K depends on the geometry and the optical constants of the prism.) This implies that the intersection point of the zero reflection curve with the optical constant curve will move by $\frac{K\Delta n_3}{\sin(\alpha)}$ in the direction of $\hat{\mathbf{t}}$ (this follows from the right angle triangle involved). Therefore, the change of the wavelength (which arises due to the change of the refractive index of sensing medium) will be $\Delta\bar{\lambda} = \frac{K\Delta n_3}{\sin(\alpha)} \hat{\mathbf{t}} \cdot \hat{\mathbf{z}}$, where $\hat{\mathbf{z}} = (0,0,1)$ is the unit vector along the dimensionless wavelength. Therefore, the sensitivity of TDM amplitude detection can be written as

$$S = \frac{\Delta\lambda}{\Delta n_3} = \frac{\lambda_0 \Delta\bar{\lambda}}{\Delta n_3} = \frac{1}{\sin(\alpha)} \frac{K\lambda_0}{\sqrt{\left(\frac{dn}{d\bar{\lambda}} \right)^2 + \left(\frac{dk}{d\bar{\lambda}} \right)^2 + 1}} = \frac{Q}{\sin(\alpha)},$$

where $Q = \frac{K\lambda_0}{\sqrt{\left(\frac{dn}{d\bar{\lambda}} \right)^2 + \left(\frac{dk}{d\bar{\lambda}} \right)^2 + 1}}$.

The formula $S = \frac{Q}{\sin(\alpha)}$ describes the dependence of TDM sensitivity on intersection angle α and was used in the main text.

Samples Fabrication

High-quality regular and homogenous arrays of gold coupled dot pairs were produced by e-beam lithography on a clean microscopic glass substrate covered by a thin Cr (5nm) sublayer (routinely used to avoid charging during electron beam lithography). We employed a double-layered resist (80 nm of 3% 495 polymethyl methacrylate (PMMA) for the bottom resist layer and 50 nm of 2% 950 PMMA for the top layer) in order to improve the subsequent lift-off process. The exposure was performed using a LEO-RAITH e-beam lithography system followed by development in 1:3 methyl isobutyl ketone (MIBK):isopropanol (IPA) developer for 30 s. After lithography, we deposited 5nm of Cr (to improve adhesion) and 90nm Au by electron beam evaporation with the help of a Moorfield system. Our deposition rate was controlled precisely at 0.1 $nm \cdot s^{-1}$ and the base pressure was 1.0×10^{-6} Torr. The thickness of growing metal film was monitored by a calibrated quartz microbalance (CQM). For the lift-off procedure, the sample was immersed in acetone for approximately 1 h. Finally, a scanning electron microscopy (SEM) image of the fabricated double nanodots structure was taken to determine the size of dots, periodicity of nanostructure and separation between dots in the pair. The fabrication of our samples is described in more detail in our previous works [1].

Ellipsometric parameters Ψ and Δ

Ellipsometry is a sensitive method that can be used to measure optical properties of materials. Ellipsometry routinely provides the amplitude (Ψ) and the phase (Δ) parameters for light reflected from an object. These parameters are related to the complex reflected field amplitudes $r_p = \frac{E_p}{E_i}$ and $r_s = \frac{E_s}{E_i}$ (where E_i is the incident light, E_p and E_s are the reflected fields for *p*- and *s*- polarizations, respectively) by the following equation $\rho = \frac{r_p}{r_s} = \tan(\Psi)\exp(i\Delta)$ [2]. The function Ψ represents the modulus of the ratio of Fresnel reflection amplitudes for *p*- and *s*-polarizations, while Δ provides the phase shift between *p*-and *s*-components of the light. A spectroscopic ellipsometer can measure the dependence of Ψ and Δ on light wavelength. In addition, a variable angle ellipsometer allows one to measure the spectral dependences of Ψ and Δ on angle of incidence. Intensity reflections and transmissions (R_p, T_p) for *p*- and (R_s, T_s) for *s*-polarized light at various angles of incidence can also be measured. The measurements of TDM nanostructures were performed across a wavelength range of 240-1700nm with the help of a variable angle spectroscopic ellipsometer (VASE) M-2000F, manufactured by J.A. Woollam, using a rotating compensator-analyzer configuration.

ATR spectroscopic ellipsometry and spectral sensitivity of samples measured with water-glycerol mixtures

Optical properties of our samples were measured using a focused beam M-2000F spectroscopic ellipsometer produced by J.A. Woollam. A pair of ellipsometric parameters Ψ and Δ were recorded in the wavelength range of 250 nm to 1700 nm with a wavelength step of around 1 nm at variable incident angles of 45–75° using an incident light beam of sizes around 30 µm × 60 µm. In our experiments, we have used attenuated reflection geometry (ATR), which is the best suited

for biosensing applications [3-5]. The ATR biosensing set-up was realised by adding a 45° glass prism and an ad-hoc micro-fluidic flow cell. The glass slide was brought into optical contact to the glass prism through the index matching glycerol layer. The spectral sensitivity of TD metamaterials shown in the main Fig. 3d were measured using the water-glycerol mixtures. The water-glycerol solutions with different index of refraction were pumped into the flow cell. The absence of air bubbles were checked by the CCD camera placed directly above the cell. The typical measurement time of one ellipsometric spectra was about 10-30s (depending on acquisition time for each spectral point).

Biosensing experiment on quantification of folic acid

Folic acid (FA, N-(4-<[(2-amino-4-oxo-1,4-dihydropteridin-6-yl)methyl]amino>benzoyl)-L-glutamic acid, vitamin B9, pteroyl-L-glutamic acid) is a critically important vitamin for human health having molecular weight of 441.4 Da. FA is a parent of a group of enzyme cofactors (referred to as folates) which play a significant role in the formation of purines, pyrimidines and methionine. FA is involved in DNA, RNA and protein biosynthesis. A lack of FA or its altered metabolism may significantly influence human health. For example, FA deficiency may lead to anaemia, psychiatric disorders, cardiovascular and cerebrovascular diseases, carcinogenesis or neuronal tube defects in newborns. Regular consumption of FA is necessary to avoid the risk of heart diseases and for pregnant women to prevent malformations of the spine, skull, and brain in newborns. The normal level of FA in health human serum is 3-20 ng/mL (6.8 – 45.3 nM), however, various reasons can cause a decrease in the FA concentration by more than 10 times. Taking into account the situation when the sample serum is limited (e.g. in newborns) and the necessity to dilute the sample to eliminate the matrix effect, the methods for FA diagnosis should be very sensitive. Therefore, precise and quantitative FA detection is critically important both for

medical diagnostics and for fundamental research concerning risks for health caused with impaired FA metabolism.

1. Chemicals

Research Centre for Molecular Diagnostics and Therapy, Russia: Mouse anti-FA IgG, clone FA1; Merck Millipore, Germany: Albumin from bovine serum, fraction V (BSA); Sigma, Germany: 2-(N-Morpholino)ethanesulfonic acid sodium salt (MES), Dimethyl sulfoxide (DMSO), N-(3-Dimethylaminopropyl)-N′-ethylcarbodiimide hydrochloride (EDC), N-Hydroxysuccinimide (NHS); Pierce, USA: BCA Protein Assay Reagent (bicinchoninic acid); GE Healthcare Life Sciences, USA: NAP-5 columns; Alfa Aesar, UK: folic acid dehydrate (FA); Dia-M, Russia: 1,4-Dithiothreitol (DTT); all other chemical reagents were of analytical grade and were used without further purification.

2. Bovine serum albumin labelling with folic acid

Bovine serum albumin labelled with folic (BSA-FA) acid was prepared as follows. Folic acid (FA) with EDC and NHS were dissolved in buffer, containing 50% DMSO and 50% of 0.1 M MES at final concentration of FA of 10 g/L with mass ratio of FA:EDC:NHS = 10:6.5:4.8. The obtained mixture was incubated for 40 min at +20 °C, after that 400 μL of BSA at 6.7 g/L were added to this mixture. The mixture was incubated for 8 h at room temperature. Then the obtained conjugate was purified from reaction by-products using NAP-5 Columns (GE Healthcare Life Sciences, USA), and the concentration of conjugate was measured using BCA protein assay according to manufacturer's recommendations.

3. Bio-functionalisation of glass slides with BSA-FA conjugate

The surface of gold nanoparticles of the array was bio-functionalised with a carrier protein (bovine serum albumin, BSA) conjugated with FA as schematically presented in Fig. 1A. For nanoparticle modification, BSA-FA (or BSA for control experiment) solution at 1 g/L was

incubated with DTT at molar ratio of BSA:DTT = 1:34 for 30 min at 70 °C in carbonate-bicarbonate buffer (4 mM Na_2CO_3, 50 mM $NaHCO_3$, pH 9.2). Then, DTT-treated protein solution at 0.5 g/L in carbonate-bicarbonate buffer was dropped onto the surface of nanoparticle array. After incubation, the surface was dried under air and rinsed with dH_2O and phosphate-buffered saline (PBS, 137 mM NaCl, 2.7 mM KCl, 4.77 mM $Na_2HPO_4 \cdot 2H_2O$, 1.7 mM KH_2PO_4, pH 7.4).

4. Detection of folic acid.

For the detection of folic acid, samples under investigation containing different FA concentrations were incubated with fixed concentration of anti-FA antibodies. Namely, BSA-FA covered nanoparticle array was sequentially exposed to 30 μg/mL anti-FA antibody in PBS with 1% BSA pre-incubated with sequentially 30-fold decreasing concentrations of FA. The obtained complexes (FA*anti-FA IgG) were pumped through a liquid cell connected with the biofunctionalized nanoparticle array. In such an assay, the highest FA concentration leads to the situation when all the antibodies are pre-blocked with analyte (FA) and antibodies binding to the gold surface is impossible, thus, the detected signal is minimal. Intermediate CAP concentration leads to the situation when significant part of antibody molecules is blocked with CAP in the sample under investigation and the signal decreased inversely proportional to the CAP concentration in a sample, or, what is the same, directly proportional to the number of free antibody molecules which are not bind with CAP in solution. In the case of minimum FA concentration, the large amount of antibody molecules can bind to the surface of gold nanodots, thus providing a substantially big detected signal which is proportional to the quantity of surface-bound antibodies. Thus, label-free competitive form of CAP detection assay was realized. One can see (main Fig. 4c) that the wavelength shift started to come to a saturation at concentrations of FA lower than 5 pM. The detected signal forms a plateau when all the sites of binding on the

surface of gold nanodots (namely, FA molecules on the protein carrier BSA) are associated with antibodies and no signal increase is possible.

5. LOD quantification.

The limit of detection (LOD) for FA in the performed competitive assay, was determined as LOD = A – 3·σ, where A is the maximal signal on the saturation, σ is the error in the measurement at the last "zero" point. Substituting the value of σ for spectral measurements (0.4 nm), we can determine that spectral LOD is equal to 0.22 nM.

References

1. Kravets, V. G.; Schedin, F.; Jalil, R.; Britnell, L.; Gorbachev, R. V.; Ansell, D.; Thackray, B.; Novoselov, K. S.; Geim, A. K.; Kabashin, A. V.; Grigorenko, A. N., Singular phase nano-optics in plasmonic metamaterials for label-free single-molecule detection. *Nature Materials* **2013,** *12* (4), 304-309.

2. Azzam, R. M. A.; Bashara, N. M., *Ellipsometry and Polarized Light*. North-Holland: Amsterdam, 1977.

3. Turbadar, T., Complete Absorption of Light by Thin Metal Films. *Proceedings of the Physical Society* **1959,** *73* (1), 40-44.

4. Raether, H., Surface plasmons. In *Springer Tracts in Modern Physics*, Springer-Verlag: 1988; Vol. 111.

5. Wu, F.; Thomas, P. A.; Kravets, V. G.; Arola, H. O.; Soikkeli, M.; Iljin, K.; Kim, G.; Kim, M.; Shin, H. S.; Andreeva, D. V.; Neumann, C.; Küllmer, M.; Turchanin, A.; De Fazio, D.; Balci, O.; Babenko, V.; Luo, B.; Goykhman, I.; Hofmann, S.; Ferrari, A. C.; Novoselov, K. S.; Grigorenko, A. N., Layered material platform for surface plasmon resonance biosensing. *Scientific Reports* **2019,** *9* (1), 20286.
